# Biatractylolide Modulates PI3K-Akt-GSK3*β*-Dependent Pathways to Protect against Glutamate-Induced Cell Damage in PC12 and SH-SY5Y Cells

**DOI:** 10.1155/2017/1291458

**Published:** 2017-09-17

**Authors:** Li Zhu, Ning Ning, Yu Li, Qiu-Fang Zhang, Yong-Chao Xie, Maida Irshad, Xing Feng, Xiao-Jun Tao

**Affiliations:** ^1^Department of Pharmacy, School of Medicine, Hunan Normal University, Changsha, Hunan 410013, China; ^2^The First Affiliated Hospital, Hunan Normal University, 61 West Jiefang Road, Changsha, Hunan 410005, China; ^3^Department of Pharmacology, Hubei University of Medicine, Shiyan 442000, China

## Abstract

Biatractylolide, isolated from the ethyl acetate extract of* Atractylodes macrocephala*, has shown various pharmacological activities such as antitumor and antioxidant activities. In this work, we aim to study the protective effect of biatractylolide on glutamate-induced rat adrenal pheochromocytoma cell (PC12) and human bone marrow neuroblastoma cell line (SH-SY5Y) injury and preliminarily explore its mechanism. The results showed that glutamate was cytotoxic with an inhibitory concentration 50% (IC50) of 8.5 mM in PC12 and 10 mM in SH-SY5Y cells. In this work, the preincubation with biatractylolide (10, 15, and 20 *μ*M) observably improved cell viability, inhibited the apoptosis of cells induced by glutamate, and reduced the activity of LDH. AO staining revealed that apoptosis of cells was decreased. Additionally, the results of western blotting manifested that pretreatment with biatractylolide could downregulate GSK3*β* protein expression and upregulate p-Akt protein expression, thereby protecting PC12 and SH-SY5Y cells from injury. All these findings indicate that biatractylolide has a neuroprotective effect on glutamate-induced injury in PC12 and SH-SY5Y cells through a mechanism of the PI3K-Akt-GSK3*β*-dependent pathways.

## 1. Introduction

Alzheimer's disease (AD) is a progressive neurodegenerative disease, used to describe a wide range of conditions, and its appearance was related to advanced age [1]. The main clinical symptoms included memory loss and cognitive impairment. At present, common clinical drugs in treatment of AD are most AChE inhibitors [2] and N-methyl-d-aspartate (NMDA) receptor antagonist [3]. Cholinesterase inhibitors can alleviate cholinergic deficit and improve neurotransmission by blocking the degradation of Ach [4, 5] and are therefore considered to be an effective and efficient way for the treatment and prevention of AD.

PC12 and SH-SY5Y cell, neural cell lines, has become an ideal cell model for exploring the mechanism of nerve cell injury in vitro and the protective effect of drugs on neuronal cells because of its typical characteristics of neuroendocrine cells [6, 7]. A large number of studies indicate [8–10] that abnormal aggregation of glutamate in the cells can lead to degeneration of the nervous system_,_ which can cause nerve excitability toxicity. Several studies have demonstrated that the main reason for the loss of neurons in the central nervous system is due to the overexcitation of glutamate receptors and high concentration of glutamate induces PC12 and SH-SY5Y cell death [11–13]. Therefore, glutamate-induced PC12 and SH-SY5Y cells have been chosen to establish nerve injury model in our study.

The roots of Atractylodis Macrocephalae Rhizoma have been widely used in traditional Chinese medicine due to its various pharmacological activities including antioxidation [14], gastroprotective [15], antitumor [16], anti-AD [17] effects. Biatractylolide is an active ingredient existing in Atractylodis Macrocephalae Rhizoma and its small molecule is double sesquiterpene ester which has a novel symmetrical structure. Our previous study confirmed that biatractylolide has a significant effect on reducing the activity of AChE in the brain and improving the memory ability of mouse dementia induced by aluminum trichloride [18]; moreover, biatractylolide can significantly reduce cholinesterase activity in model rats and improve the behavior and memory of AD model rats induced by A*β*_1−40_ [19]. Importantly, biatractylolide can inhibit the enzyme activity of AChE and downregulate the expression of AChE in a dose-dependent manner, which has been confirmed in our previous study [20]. However, the specific mechanisms of biatractylolide are not well understood, and there are no reports about protective effect of biatractylolide currently. Therefore, in our study, biatractylolide was applied to explore its protective effects against the glutamate-induced cell damage in PC12 and SH-SY5Y cells and mechanisms, providing a theoretical basis for its great potential application on the treatment of Alzheimer's disease.

## 2. Materials and Methods

### 2.1. Reagents

**Figure 1:**
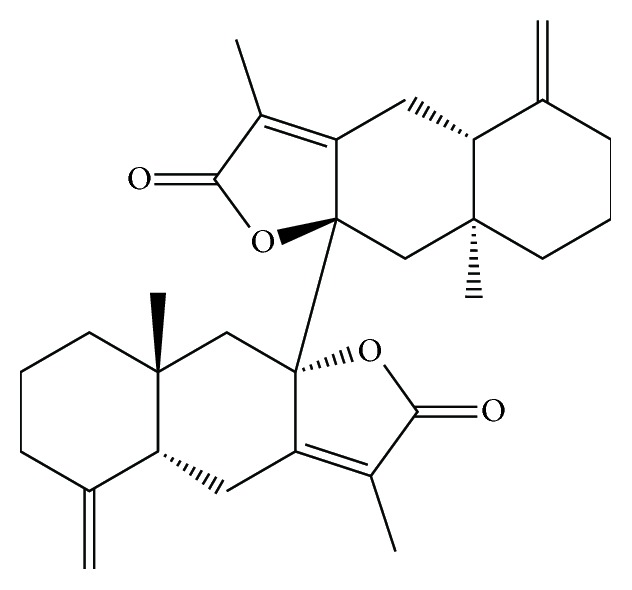
*The chemical structure of biatractylolide*. Biatractylolide is a kind of internal symmetry double sesquiterpene novel ester compound.

Biatractylolide was isolated from the ethyl acetate extract of* Atractylodes macrocephala* by multistep chromatographic processing [20] (Figure 1). Glutamate and DMSO were purchased from Sigma Chemicals (USA). Antibodies for the protein characteristics were against total Akt, phosphor-Akt, total GSK3*β*, phosphor-GSK3*β*, and *β*-actin which were purchased from Cell Signaling Technology (Danvers, MA, USA).

### 2.2. Cell Culture

PC12 cells and SH-SY5Y cells donated by Dr. James R. Woodgett (China Pharmaceutical University, Nanjing, China) were routinely cultured in DMEM medium in the presence of 10% FBS and 1% double antibody. All cells were cultured at 37°C in a humidified 5% CO_2_ incubator.

### 2.3. Experimental Grouping and Drug Treatment

By adding different concentrations of glutamate ranging from 0 to 30 mM in the preliminary experiment, we selected the concentrations (7.5 mM and 10 mM) as the most optimal injured concentrations by determining dose-response curves. The experiment was as follows: (1) blank control group; (2) glutamate injury model group; (3) drug treatment group (low, medium, and high).

### 2.4. Cell Viability Assay

1 × 10^4^ cells were seeded in 96-well culture plates and cultured overnight. Then the cells were treated with various concentrations of biatractylolide for 30 min before glutamate treatment for 24 hours in incubator. The medium was washed off with PBS and 20 *μ*L MTT Reagent was added to each well and the plate was incubated at 37°C for 4 h. Finally, the medium was removed from each well and 150 *μ*l of DMSO was added to dissolve formazan crystals. The product was quantified by measuring absorbance at 490 nm using a Dynatech MR5000 plate reader.

### 2.5. Assessment of Apoptosis

6 × 10^5^ PC12 cells and 8 × 10^5^ SH-SY5Y cells were seeded in 6-well plates and cultured overnight. Then the cells were treated with various concentrations of biatractylolide for 30 min before glutamate treatment for 24 hours in incubator. Then treated cells were digested and washed twice with cold PBS. The cell precipitation was resuspended and added to 5 *μ*L FITC Annexin V and 10 *μ*l PI and then incubated for 15 min at room temperature in dark. After adding 300 *μ*L of binding buffer, labeled cells were counted by flow cytometry within 1 h. All early apoptotic cells and necrotic/late apoptotic cells as well as living cells were detected by FACSCalibur flow cytometer and subsequently analyzed by Cell Quest software (Becton Dickinson).

### 2.6. Acridine Orange/Ethidium Bromide Staining

6 × 10^5^ PC12 cells and 8 × 10^5^ SH-SY5Y cells were seeded in 6-well plates and cultured overnight. Then the cells were treated with various concentrations of biatractylolide for 30 min before glutamate treatment for 24 hours in incubator. The treated cells were washed with cold PBS twice and fixed with formaldehyde for 30 min according to AO/EB staining assay kit instructions. Finally, they were incubated with AO (6 *μ*g/ml) for 10 min at room temperature in the dark and then observed by fluorescence microscopy.

### 2.7. Determination of LDH Activity

5 × 10^5^ PC12 cells and 6 × 10^5^ SH-SY5Y cells were seeded in 24-well plates and cultured overnight. Then the cells were treated with various concentrations of biatractylolide for 30 min before glutamate treatment for 24 hours in incubator. Twenty-four hours later, the supernatant was collected and centrifuged at 3500 rpm for 3 min and the OD value of LDH in the medium was determined by colorimetric method according to LDH assay kit.

### 2.8. Measurement of Intracellular ROS

6 × 10^5^ PC12 cells and 8 × 10^5^ SH-SY5Y cells were seeded in 6-well plates and cultured overnight. Then the cells were treated with various concentrations of biatractylolide for 30 min before glutamate treatment for 24 hours in incubator. Next, the treated cells were washed with cold PBS twice and fixed with formaldehyde for 30 min according to ROS assay kit instructions. The intracellular ROS was observed by fluorescence microscopy.

### 2.9. Detection of Mitochondrial Membrane Potential by Rhodamine-123 Staining

5 × 10^5^ PC12 cells and 6 × 10^5^ SH-SY5Y cells were seeded in 24-well plates and cultured overnight. Then the cells were treated with various concentrations of biatractylolide for 30 min before glutamate treatment for 24 hours in incubator. The treated cells were harvested and resuspended in the medium and, finally, incubated with Rhodamine-123 (10 *μ*g/ml) for 10 min at room temperature in the dark and then observed by fluorescence microscopy.

### 2.10. Western Blotting

Western blotting assay was performed using methods described by Su et al. [21]. Primary antibody was added in BSA and allowed to incubate overnight at 4°C, washed with TBS/0.05% Tween-20 for 5 times (10 min per time) before the secondary antibody was added and then incubated for an additional hour at room temperature. The membrane was again washed 3 times before adding Pierce Super Signal chemiluminescent substrate (Rockford, IL, USA) and then immediately imaged on ChemiDoc (Bio-Rad, Hercules, CA, USA). The films were scanned using EPSON PERFECTION V500 PHOTO and quantified by Image J (NIH, Bethesda, MD, USA).

### 2.11. Statistical Analysis

All data are presented as mean ± SEM and GraphPad Prism statistical software was used for analysis. Comparison between the two groups using Student's *t*-test and the variance analysis was performed with One-Way ANOVA method and statistical significance was assumed at a value of *p* < 0.05 or less.

## 3. Results

### 3.1. The PC12 and SH-SY5Y Cell Proliferation

**Figure 2:**
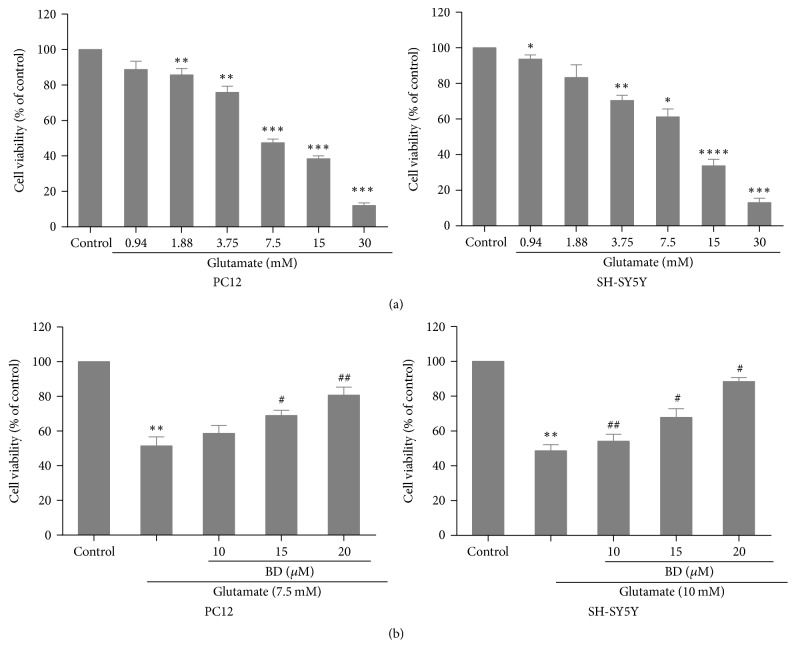
*Protective effect of biatractylolide on the viability of PC12 and SH-SY5Y cells insulted by glutamate*. (a) Viability change of PC12 and SH-SY5Y cells treated by glutamate under different concentration circumstances after 24 h. (b) Biatractylolide inhibited glutamate-induced injury in PC12 and SH-SY5Y cells. ^*∗*^*p* < 0.05, ^*∗∗*^*p* < 0.01, ^*∗∗∗*^*p* < 0.005, and ^*∗∗∗∗*^*p* < 0.001 versus control group; ^#^*p* < 0.05 and ^##^*p* < 0.01 versus model group.

Initially, we explore the effects of different concentrations of glutamate (0.94, 1.88, 3.75, 7.5, 15, and 30 mM) on the proliferation of SH-SY5Y cells and PC12 cells using MTT test. The result indicated that the inhibition rate of 7.5 mM glutamate-treated PC12 cells reached 48.2 ± 1.5% (*p* < 0.001) and 15 mM glutamate-treated SH-SY5Y cells led to 39.12 ± 2.1% decrease of cell viability (Figure 2(a)). Thus, glutamate at 7.5 mM and 10 mM was selected as model concentration. To investigate the impact of biatractylolide on cell damage induced by glutamate, we evaluated cell viability using the MTT approach. The cells were treated with various concentrations of biatractylolide for 30 min before glutamate treatment for 24 h. The result showed that biatractylolide led to a dose-dependent increase on PC12 and SH-SY5Y cells proliferation (Figure 2(b)).

### 3.2. Assessment of Apoptosis

**Figure 3:**
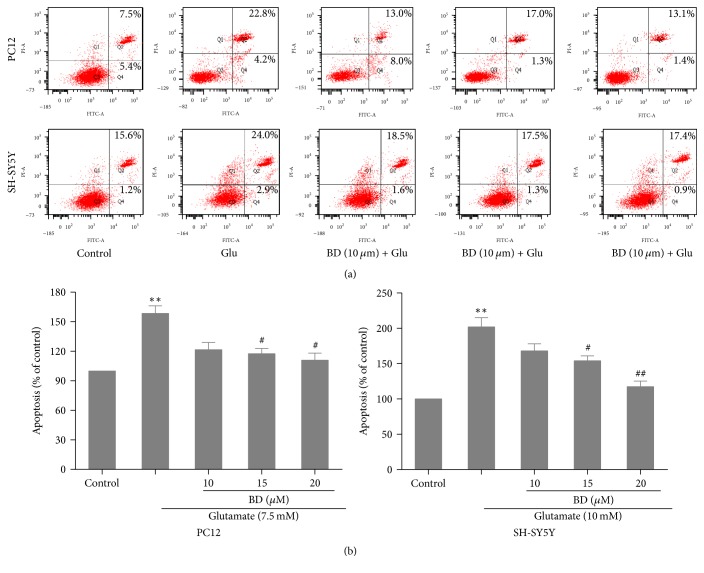
*Biatractylolide protected PC12 and SH-SY5Y cells from apoptosis induced by glutamate*. (a) Representative flow cytometry scatter plots. (b) Bar chart shows quantitative data of flow cytometry experiments. ^*∗∗*^*p* < 0.01 versus control group; ^#^*p* < 0.05 and ^##^*p* < 0.01 versus model group.

Glutamate-induced cell apoptosis was quantitatively tested by using FITC and PI. As shown in Figures 3(a)-3(b), we found that cell apoptosis rate was considerably increased to 162.8 ± 1.7% and 202.5 ± 3.2% in the model group compared to the control group; however, in the treated group, biatractylolide distinctly decreased glutamate-induced cell apoptosis in a dose-dependent fashion (*p* < 0.05).

### 3.3. Acridine Orange/Ethidium Bromide Staining

**Figure 4:**
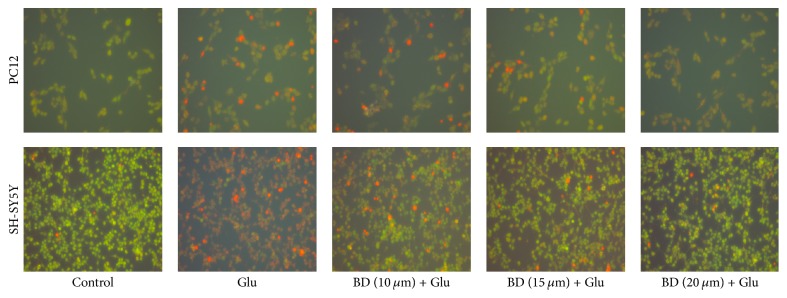
*Cell morphology of PC12 and SH-SY5Y cells stained with acridine orange (×400)*. The two cell lines were treated with various concentrations of biatractylolide for 30 min before glutamate treatment for 24 hours. The cell morphology was observed under fluorescence microscope.

We observed the morphological characteristic of apoptotic cells using the AO/EB staining test. And Figure 4 displayed that morphological changes including chromatic agglutination, karyopyknosis, and nuclear fragmentation could be observed in glutamate model group by fluorescence microscopy. Compared with the model group, the addition of different concentrations of biatractylolide can markedly inhibit cell damage and improve cell morphology in a concentration-dependent manner.

### 3.4. Determination of LDH Activity

**Figure 5:**
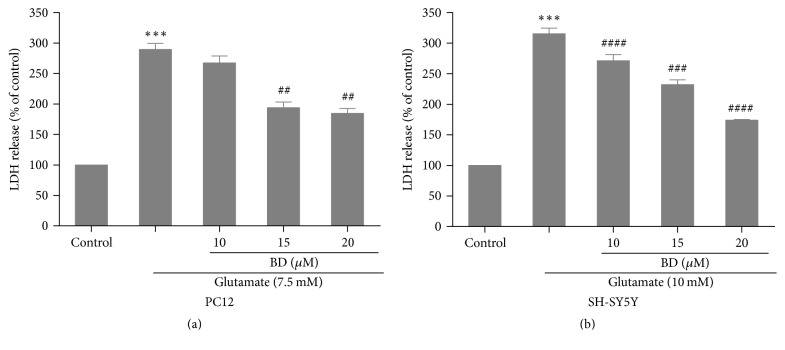
*Determination of LDH activity*. (a)-(b) After the two cell lines were treated with various concentrations of biatractylolide for 30 min before glutamate treatment for 24 hours, the LDH activity was examined by LDH assay kit. ^*∗∗∗*^*p* < 0.01 versus control group; ^##^*p* < 0.01, ^###^*p* < 0.005, and ^####^*p* < 0.001 versus model group.

We next determined the effects of biatractylolide on LDH activity in PC12 cells and SH-SY5Y cells; we found that the model group has an increase of LDH release, being 280.3 ± 0.3% and 318.22 ± 0.1% as compared to control cells (*p* < 0.01; Figures 5(a)-5(b)). However, preincubation with biatractylolide at the concentration of 15 *μ*M and 20 *μ*M significantly obstructed LDH release in the PC12 cells, which was decreased from 180.5 ± 0.9% to about 195.3 ± 2.1% as compared to model group (*p* < 0.01; Figure 5(a)). In addition, biatractylolide (10 *μ*M, 15 *μ*M, and 20 *μ*M) distinctly decreased LDH activity of SH-SY5Y cells, being 174.2 ± 0.4% (*p* < 0.001), 243.6 ± 0.1% (*p* < 0.005), and 272.1 ± 2.9% (*p* < 0.001) as compared to model group (Figure 5(b)).

### 3.5. Measurement of Intracellular ROS

**Figure 6:**
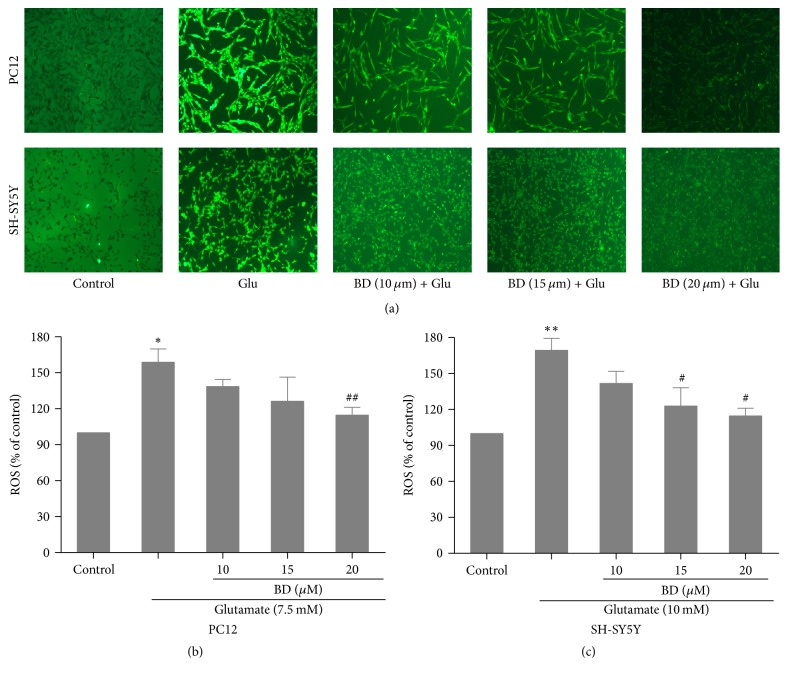
*The glutamate-stimulated ROS was effectively attenuated by biatractylolide in PC12 and SH-SY5Y cell lines*. After the two cell lines were treated with various concentrations of biatractylolide for 30 min before glutamate treatment for 24 hours, the ROS production was detected by ROS kit. (a) The fluorescence image. (b)-(c) Bar chart shows quantitative data. ^*∗*^*p* < 0.05 and ^*∗∗*^*p* < 0.01 versus control group; ^#^*p* < 0.05 and ^##^*p* < 0.05 versus model group.

We also investigated the effects of various concentrations of biatractylolide on the release of ROS in SH-SY5Y cells and PC12 cells. As shown in Figure 6(a), we found that the model group has an increase of relative fluorescence unit in PC12 and SH-SY5Y cells as compared to control cells. However, pretreatment with biatractylolide at the concentration of 20 *μ*M significantly inhibited relative fluorescence intensity of ROS to 112.6 ± 0.7% as compared to model group (*p* < 0.01) (Figure 6(b)). In addition, biatractylolide (10 *μ*M, 15 *μ*M, and 20 *μ*M) distinctly decreased ROS production of SH-SY5Y cells, being 161.2 ± 1.3% (*p* < 0.05), 133.6 ± 2.9% (*p* < 0.01), and 105.9 ± 3.4% (*p* < 0.01) as compared to model group (Figure 6(c)).

### 3.6. Detection of Mitochondrial Membrane Potential by Rhodamine-123 Staining

**Figure 7:**
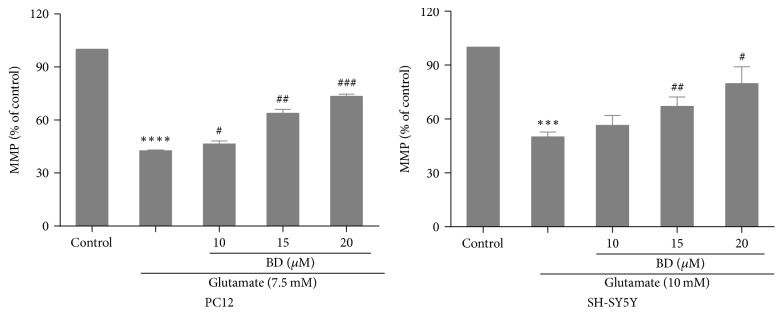
*MMP suppressed by glutamate-insult in PC12 and SH-SY5Y cell lines was effectively ameliorated by biatractylolide*. After the two cell lines were treated with various concentrations of biatractylolide for 30 min before glutamate treatment for 24 hours, the mitochondrial membrane potential was examined using the Rhodamine-123. ^*∗∗∗*^*p* < 0.005 and ^*∗∗∗∗*^*p* < 0.001 versus control group; ^#^*p* < 0.05, ^##^*p* < 0.01, and ^###^*p* < 0.005 versus model group.

As can be seen in Figure 7, after being treated with 7.5 and 10 mM glutamate, the MMP were considerably decreased compared to the control group (*p* < 0.001). Pretreatment with biatractylolide (10 *μ*M, 15 *μ*M, and 20 *μ*M) could significantly protect PC12 and SH-SY5Y cells from the glutamate-induced reduction of MMP.

### 3.7. Protein Characterization

**Figure 8:**
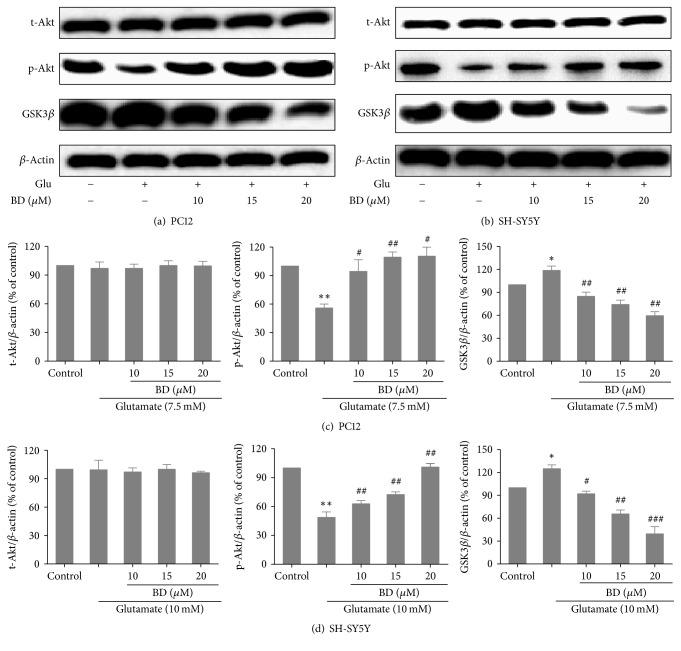
*Effects of biatractylolide on Akt and Gsk3β expressions of PC12 and SH-SY5Y cells induced by glutamate*. (a)-(b) represent protein characterization of Akt and Gsk3*β* after PC12 and SH-SY5Y cells were treated with various concentrations of biatractylolide for 30 min before glutamate treatment for 24 hours. Blots were also probed for *β*-actin as loading controls. (c)-(d) showed the ratio of different proteins to *β*-actin was calculated by the band density of each cell line using Image J software. ^*∗*^*p* < 0.05 and ^*∗∗*^*p* < 0.01 versus control group; ^#^*p* < 0.05, ^##^*p* < 0.01, and ^###^*p* < 0.005 versus model group.

PI3K-Akt signaling pathway is essential for the survival of neurons and the effect of PI3K-Akt-GSK3*β* signal transduction pathway on GSK3*β* is most important in the regulation of apoptosis. Studies have revealed that activation of PI3K-Akt-GSK3*β* pathway can have neuroprotective effects [22]. To validate whether this pathway was involved in protective effects of biatractylolide on glutamate-induced PC12 cells and SH-SY5Y cells, we further analyze protein characterization using western blotting. Figures 8(a)–8(d) showed that the expression of GSK3*β* was observably increased after glutamate treatment compared with control group (*p* < 0.05) and pretreatment with biatractylolide led to marked decreases in level of GSK3*β* of compared to the model group. In comparison, treatment of cells with glutamate induced a significant decrease in the level of p-Akt compared to control group (*p* < 0.01) and pretreatment with biatractylolide led to significant increases in level of p-Akt compared to the model group.

## 4. Discussion

Natural drug has been widely used in the development and research of neuroprotective drugs because of its low side effects, multiple targets, and high efficiency. Some studies have reported that biatractylolide could slow down the isolated guinea pig right atrium heart rate and reduce shrinkage force [23]. Furthermore, this effect could be offset by atropine, indicating that biatractylolide might inhibit cholinesterase effect. However, studies of biatractylolide in neurodegenerative diseases are rare currently; thus, the present study evaluated the neuroprotective effect of biatractylolide against glutamate-induced cell injury and its underlying mechanism. MTT assay is a common method to detect the number of viable cells. In our study, different concentrations of biatractylolide significantly increased the survival of PC12 and SH-SY5Y cells in a dose-dependent fashion. When the cell membrane was damaged, the intracellular LDH was released into the culture medium, and the content of LDH was an important index to detect the cell death. In this study, we found that the level of LDH was significantly increased in the model group, while the LDH level in the supernatant was significantly reduced in a concentration-dependent manner after treating with different concentrations of biatractylolide.

Glutamate-induced neurotoxicity may be caused by glutamate and NMDA receptors, which could produce oxidative damage, thus leading to apoptosis [24]. Acridine orange (AO) staining and PI staining [25–27] are important methods for detecting apoptosis. In our study, PI staining and acridine orange staining showed that biatractylolide could reduce the apoptosis of PC12 and SH-SY5Y cells induced by glutamate. We also found that some proportion of necrotic cells appeared in SH-SY5Y cells line, while it was not apparent in the PC12 cell line. We think that the reason for these differences may be that the sensitivity of the two cell lines to glutamate is different but the difference is reasonable and inevitable. So we conclude that biatractylolide has a protective effect on glutamate-induced apoptosis.

Oxidative stress refers to the imbalance of generation and clearance of oxygen free radicals in the body, leading to oxidative damage caused by accumulation of active oxygen and active nitrogen in the body. Numerous studies have shown that there are increases in the level of ROS in neurodegenerative diseases [28, 29], which is implicated in glutamate-induced neurotoxicity and the progression of AD, so the ROS assay kit was used to detect the release of intracellular ROS in this study. Biatractylolide significantly increased the ROS levels compared to the model group in our study, which indicated that biatractylolide significantly reduced the production of glutamate-induced ROS.

Rh123 [30, 31] is a lipophilic cationic fluorescent dye that is permeable to cell membranes and can be selectively enriched in mitochondria. When the cells are in the survival state, Rh123 is accumulated in mitochondria and emits green fluorescence through the cell membrane, but in apoptosis, the ability of cell mitochondria to accumulate Rh123 was lost and the fluorescence intensity was decreased. In this study, the ROS and MMP results suggest that biatractylolide may have neuroprotective effects.

The process of glutamate-induced nerve cell damage is related to multiple signal transduction pathways. Numerous studies show that PI3K-Akt-GSK3*β* signal transduction pathway plays important roles in the neuroprotective process [32, 33]. Our present study demonstrated that the two cell lines were exposed to glutamate, the levels of p-Akt decreased, and GSK3*β* increased significantly compared with the control group while the level of t-Akt was almost unchanged. After treating with biatractylolide, the levels of p-Akt increased and GSK3*β* decreased in a concentration-dependent manner, suggesting that these changes were abolished by pretreatment with biatractylolide and biatractylolide may play a neuroprotective role by activating PI3K-Akt-GSK3*β* pathway.

As we mentioned above, biatractylolide effectively protects PC12 and SH-SY5Y cells from the glutamate-induced neuronal damage and it is suggested that the mechanism of protective effect of biatractylolide may be mainly related to PI3K-Akt-GSK3*β* pathway.
